# An Aged Canid with Behavioral Deficits Exhibits Blood and Cerebrospinal Fluid Amyloid Beta Oligomers

**DOI:** 10.3389/fnagi.2018.00007

**Published:** 2018-01-30

**Authors:** Clare Rusbridge, Francisco J. Salguero, Monique Antoinette David, Kiterie M. E. Faller, Jose T. Bras, Rita J. Guerreiro, Angela C. Richard-Londt, Duncan Grainger, Elizabeth Head, Sebastian G. P. Brandner, Brian Summers, John Hardy, Mourad Tayebi

**Affiliations:** ^1^Department of Pathology and Infectious Diseases, Faculty of Health and Medical Sciences, University of Surrey, Guildford, United Kingdom; ^2^Fitzpatrick Referrals, Godalming, United Kingdom; ^3^School of Medicine, Western Sydney University, Campbelltown, NSW, Australia; ^4^School of Veterinary Medicine, College of Medical, Veterinary, and Life Sciences, University of Glasgow, Glasgow, United Kingdom; ^5^Department of Molecular Neuroscience, Institute of Neurology, University College London, London, United Kingdom; ^6^Department of Medical Sciences and Institute of Biomedicine - iBiMED, University of Aveiro, Aveiro, Portugal; ^7^Division of Neuropathology and Department of Neurodegenerative Disease, Institute of Neurology, University College London, London, United Kingdom; ^8^Department of Pharmacology and Nutritional Sciences, Sanders Brown Center on Aging, University of Kentucky, Lexington, KY, United States; ^9^Retired, Campbelltown, NSW, Australia

**Keywords:** canine cognitive dysfunction, Alzheimer, canine, Aβ oligomers, neuropathology, aggregation, neurotoxicity

## Abstract

Many of the molecular and pathological features associated with human Alzheimer disease (AD) are mirrored in the naturally occurring age-associated neuropathology in the canine species. In aged dogs with declining learned behavior and memory the severity of cognitive dysfunction parallels the progressive build up and location of Aβ in the brain. The main aim of this work was to study the biological behavior of soluble oligomers isolated from an aged dog with cognitive dysfunction through investigating their interaction with a human cell line and synthetic Aβ peptides. We report that soluble oligomers were specifically detected in the dog's blood and cerebrospinal fluid (CSF) via anti-oligomer- and anti-Aβ specific binders. Importantly, our results reveal the potent neurotoxic effects of the dog's CSF on cell viability and the seeding efficiency of the CSF-borne soluble oligomers on the thermodynamic activity and the aggregation kinetics of synthetic human Aβ. The value of further characterizing the naturally occurring Alzheimer-like neuropathology in dogs using genetic and molecular tools is discussed.

## Introduction

Aging dogs spontaneously deposit human-type amyloid β (Aβ) peptide (Selkoe et al., 1987; Johnstone et al., 1991) and thus are a natural higher mammalian model of aging. The canine Aβ precursor protein (APP) is virtually identical to human APP (~98% homology). In parallel with progressive Aβ pathology, aged dogs show decline in measures of learning and memory that are correlated with the extent and location of Aβ (Cotman and Head, 2008). However, a recent report by Borghys and colleagues demonstrated that high levels of Aβ in the cerebrospinal fluid (CSF) of young and middle-aged dogs correlated with impaired learning (Borghys et al., 2017), in contrast with previous reports showing that CSF Aβ content decreases in the aged/aging dog (Head et al., 2010). Of note, cognitive decline occurs prior to the accumulation of Aβ plaques in the canine brain, suggesting that earlier assembly states of Aβ (e.g., oligomers, protofibrils) may be the toxic species in the canine brain as in the human brain (Head et al., 2010).

In this study, we describe the presence of Aβ soluble oligomers in serum and CSF of a 12-year-old Samoyed (referred to as “the Subject” throughout this report). Upon neurological examination, this subject displayed signs of cognitive impairment and magnetic resonance imaging (MRI) showed diffuse cortical atrophy. Aβ immunostaining demonstrated extensive diffuse plaques in the neocortex and hippocampal regions; but no tau staining. Of importance, CSF and serum from this subject exhibited neurotoxic effects following treatment of a human neuroblastoma cell line and led to efficient aggregation of synthetic human Aβ peptides.

## Case report

### Ethics statement

This project was reviewed by the University of Surrey Ethics Committee and verified that the aspects of the study pertaining to the Samoyed dog including use of body fluids excess and post mortem material did not come under the auspices of Animals (Scientific Procedures) Act 1986 (ASPA). The subject's owner gave informed consent to participation in the study. Full clinical and neurological examination and presentation, including MR imaging assessment are found in Supplementary Materials. Negative control CSF was obtained from a 10-year-old male Rottweiler suffering from nodular granulomatous episclerokeratitis following submission for routine teaching post-mortem and not subject to animal ethics guidelines. CSF derived from a 79-year-old patient with advanced sporadic AD (pos1-CSF) and from a 65 year old patient with advanced sporadic AD (pos2-CSF) were provided by The UK Multiple Sclerosis Tissue Bank.

### Clinical presentation

**Figure 1:**
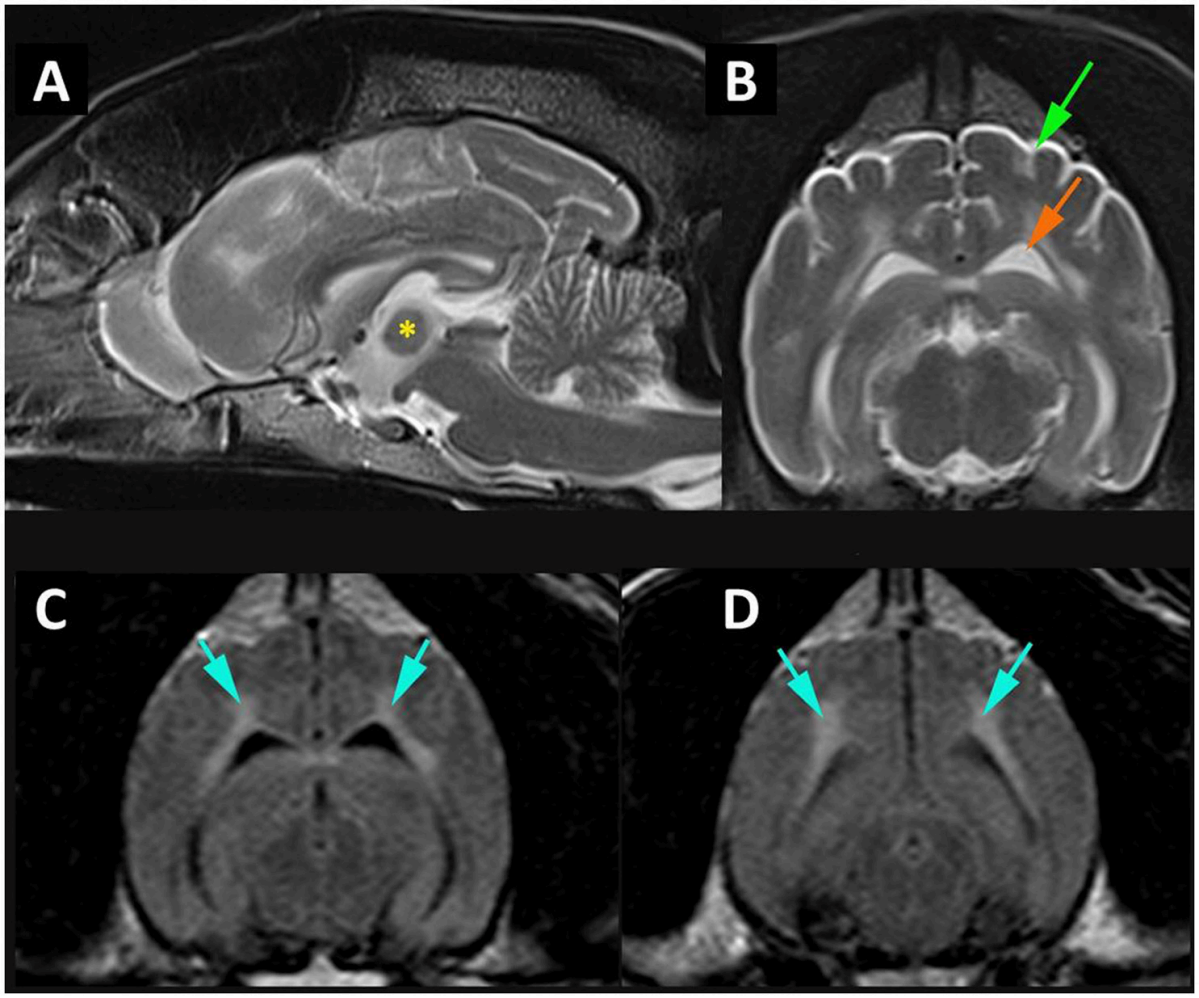
MR imaging of cortical atrophy and myelin degeneration. **(A)** Mid-sagittal T2W image of the brain demonstrating atrophy of the intra-thalamic adhesion (^*^) height 4.76 mm. **(B)** Transverse T2W at the level of temporal lobes demonstrating cortical atrophy with widening of the subarachnoid space (green arrow) and enlargement of the lateral ventricle (orange arrow). **(C)** Transverse FLAIR at level of temporal lobes and **(D)** transverse FLAIR at level of occipital lobes and midbrain demonstrating white matter hyperintensity (blue arrows).

A 12-year-old neutered male Samoyed dog was presented for pain management and evaluation of difficulty in rising. Neurological examination revealed tetraparesis and reduced spinal reflexes and muscle tone consistent with a polyneuropathy. The difficulty rising was attributed to this, complicated by the sedative polypharmacy. The historical and consulting room behavior suggested a cognitive function deficit possibly complicated by a urinary tract infection. The brain MRI scan revealed a diffuse cortical atrophy and a hyperintensity in the white matter on T2W, particularly in the corona radiata (Figure 1); changes consistent with age-associated cognitive decline (Hasegawa et al., 2005). Signs were controlled for the next 6 months after which the dog deteriorated and described as being extremely agitated and distressed during the night which was unresponsive to changing dose of medication and resulting in significant sleep deprivation for the owners. A full post mortem examination was undertaken at the pathology facility at the University of Surrey and the brain and other organs were removed for further analysis. A more extensive description of the clinical and neurological examination and presentation is included as supplementary results.

### Detection of Aβ species in the subject's serum, CSF, and brain

**Figure 2:**
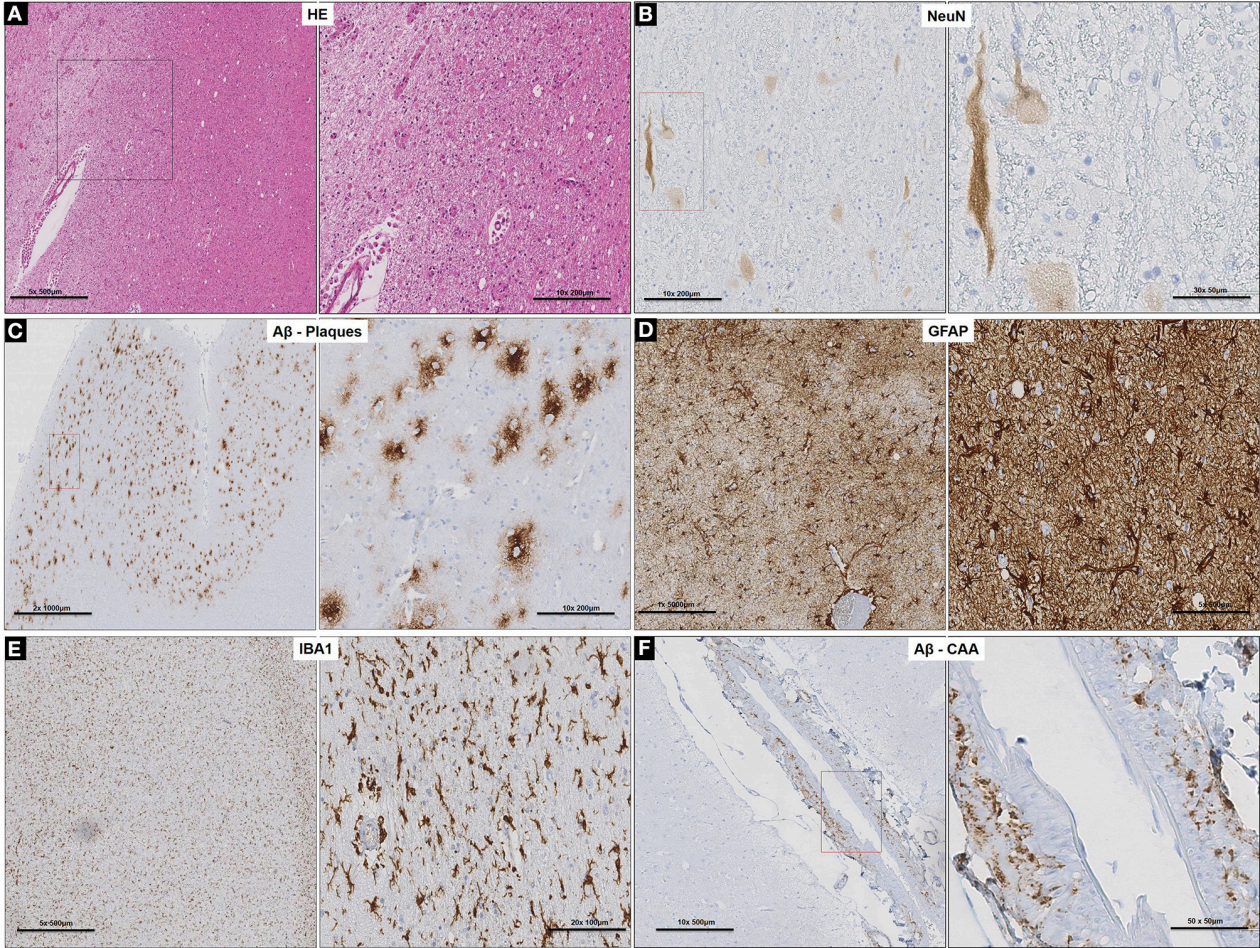
Cortico-neuropathological microscopic lesions. **(A)** Cortical degeneration (vacuolation) and neuronal death observed on routine H&E stained sections of prefrontal cortex and diffuse cerebral periventricular white matter degeneration (vacuolation and pallor); **(B)** Neuronal degeneration and loss (arrows) confirmed with neuron-specific marker, NeuN. **(C)** Specific labeling of diffuse Aβ plaques with anti-Aβ specific antibody in the prefrontal cortex. **(D)** Extensive gliosis in prefrontal cortex revealed by GFAP stain and associated with **(E)** microglia activation demonstrated with Iba1 staining. **(F)** Specific labeling of CAA with anti-Aβ specific antibody in the cortical blood vessels in the prefrontal cortex.

Neuronal loss and degeneration was marked in the cortical region of the subject (Figures 2A,B) while intense binding of large extra-cellular diffuse Aβ plaques, recruitment, and activation of astrocytes and microglial cells (Figures 2C–E) were observed in the neocortex and hippocampus. Pronounced cerebral amyloid angiopathy (CAA) was observed in several cortical blood vessels (Figure 2F), but was less intense in the tunica media of leptomeningeal arteries. Of note, PHF and the signaling adapter p62 were not detected (data not shown) (Babu et al., 2005). Furthermore, white matter degeneration took the form of myelin vacuolation and isomorphic gliosis (Figure 2A) with macrophages containing pale yellow cytoplasmic material evident as small clusters of cells and as perivascular aggregates (Figure 2A). Iba1 stain demonstrated more widespread microglial/macrophage activation while GFAP confirmed the brisk gliosis (data not shown).

**Figure 3:**
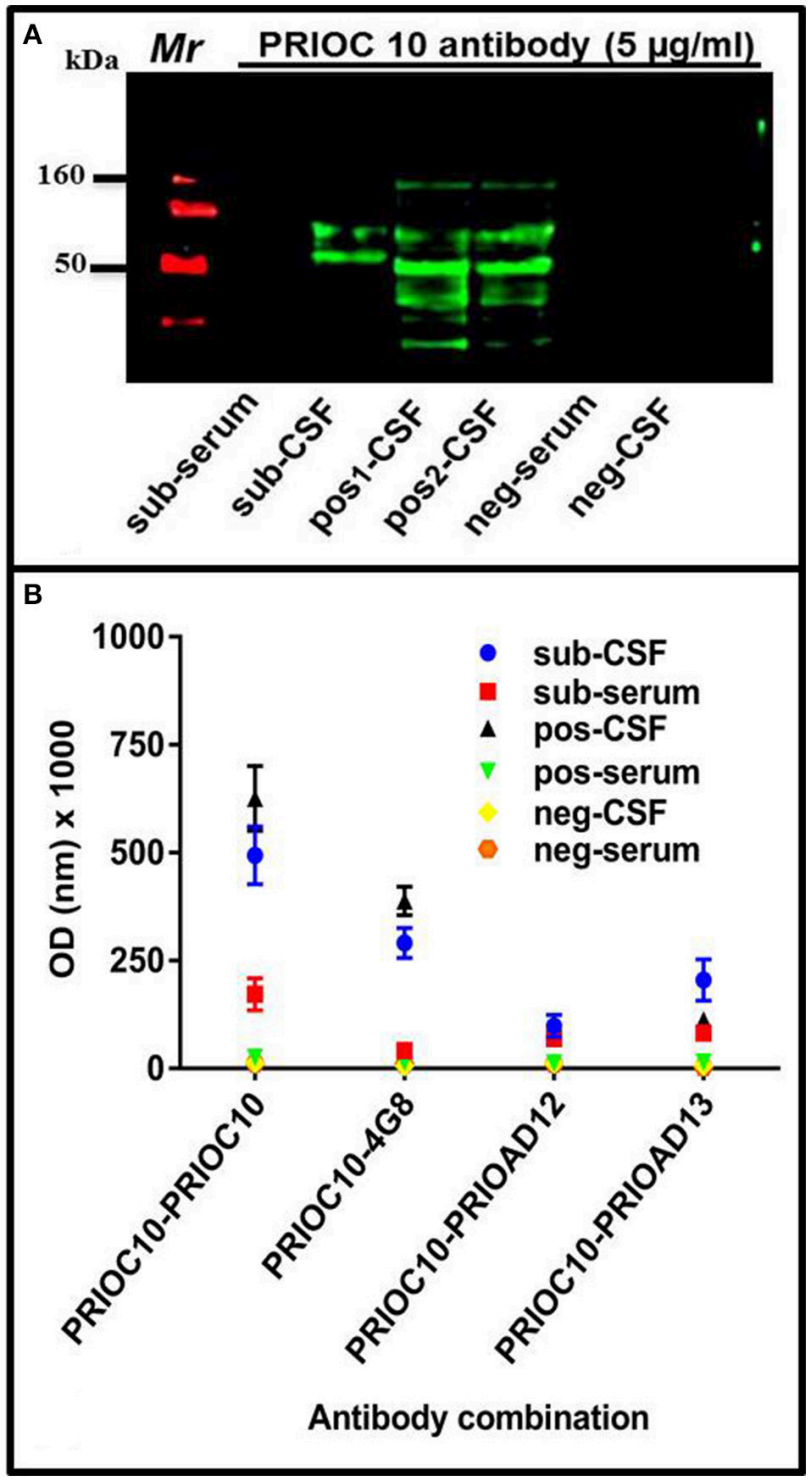
Immuno-detection of Aβ soluble oligomers. **(A)** Western blot showing the ability of PRIOC10 anti-oligomer antibody to strongly immunocapture precipitates from the CSF (sub-CSF) but not the serum (sub-serum) derived from the dog. Positive control derived from CSF from Alzheimer's disease patients (patient case number 51486—age 79 years [pos1-CSF] and 60649–65 years [pos2-CSF]) displayed PRIOC10-positivity for soluble oligomers. In contrast, CSF (neg-CSF) and serum (neg-serum) derived from a Rottweiler failed to display PRIOC10 labeling on Western blotting. **(B)** A customized Sandwich ELISA was used to detect specific Aβ oligomers. Biotinylated anti-Aβ 4G8 (Aβ) (Thakker et al., 2009), PrioAD12 (Aβ_1–40_) or PrioAD13 (Aβ_1–42_) (David et al., 2014) were used to immunocapture Aβ species contained in the CSF (sub-CSF) and the serum (sub-serum) precipitates derived from the dog. Purified PRIOC10 anti-oligomer antibody was added to immunodetect specifically Aβ soluble oligomers. The sandwich format of the assay has established the specificity of PRIOC10 antibody for Aβ oligomers. Values shown are the mean Aβ ± SD from 12 observations.

The ability of PRIOC10 mAb to bind to Aβ soluble oligomers in sub-serum and sub-CSF was assessed by Western blotting and Sandwich ELISA. Western blotting results indicate that PRIOC10 mAb bound to soluble oligomers in sub-CSF but not in sub-serum or negative control CSF (neg-CSF) derived from the Rottweiler (Figure 3A). Further, PRIOC10 mAb pattern of recognition revealed several bands ranging between 50 and 160 kDa (Figure 3A); and positive control CSF (pos-CSF) isolated from two patients with AD also led to detection of soluble oligomers (Figure 3A).

We then set out to investigate the specificity of the soluble oligomers and confirm that the PRIOC10-specific bands detected in the sub-CSF were mainly composed of Aβ using our customized sELISA as described previously (Tayebi et al., 2011). PRIOC10, 4G8 (Aβ) (Thakker et al., 2009), PrioAD12 (Aβ_1–40_), or PrioAD13 (Aβ_1–42_) (David et al., 2014) were used as immunocapture antibodies to detect soluble oligomers in the sub-CSF and sub-serum, followed by immunodetection with biotinylated PRIOC10 (Figure 3B). We show that the 4G8 vs. biot-PRIOC10 combination detected highest levels of Aβ in sub-CSF as expressed in OD-values (*p* = 0.0001) and almost matched CSF levels detected with the PRIOC10 vs. biot-PRIOC10 combination. This was followed by the PRIOAD13 vs. biot-PRIOC10 combination that displayed higher CSF levels of detection compared with the PRIOAD12 vs. biot-PRIOC10 combination (*p* = 0.0279). Of note, sELISA lead to detection of Aβ in the sub-serum (Figure 3B) in contrast with western blotting, albeit with significantly lower OD intensity as compared to levels detected in the sub-CSF.

### Mutations in presenilin 1, presenilin 2, and amyloid precursor protein genes were not identified

Genome assembly CanFam3.1 and transcripts ENSCAFT00000013599.4 for *APP*, ENSCAFT00000026626.1 for *PSEN1*, and ENSCAFT00000025451.3 for *PSEN2* were used for primer design (See Supplementary Results: Table S2) and as the reference for sequence analysis. The subject's DNA was used to sequence the genes that when mutated are known to cause AD in humans. No variants expected to be pathogenic were identified. Synonymous variants were found in *APP* (p.G120G; p.K178K; p.A242A; p.T266T), and *PSEN2* (p.P436P).

### CSF and serum derived from subject was toxic to neuron-like SH-SY5Y cell line

**Figure 4:**
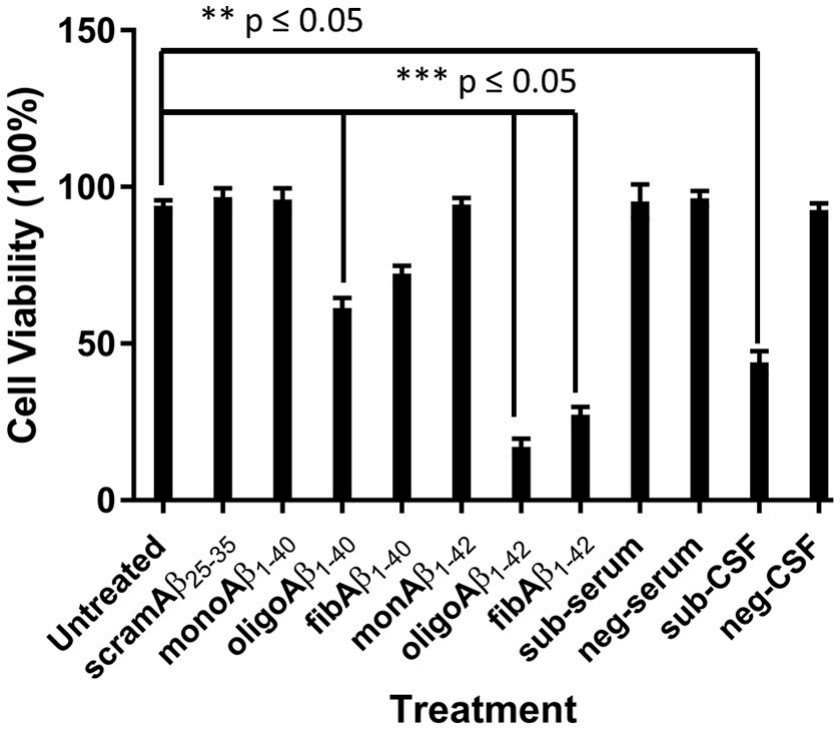
CSF but not serum derived from the aged dog leads to neurotoxicity of neuron-like SH-SY5Y cell line. The effect of CSF and serum on the survival of SH-SY5Y cell line was compared with monoAβ_1–40_, monoAβ_1–42_, scramAβ_25–35_, oligoAβ_1–40_, oligoAβ_1–42_, fibAβ_1–40_, fibAβ_1–42_ as well as CSF (neg-CSF) and serum (neg-serum) derived from a Rottweiler. Values shown are the mean cell survival ± SD from 12 observations.

The toxic effects of monoAβ_1–40_, monoAβ_1–42_, scramAβ_25–35_, oligoAβ_1–40_, oligoAβ_1–42_, fibAβ_1–40_, fibAβ_1–42_, sub-serum, and sub-CSF on differentiated human neuroblastoma cell line RA-SH-SY5Y viability was investigated using the MTT assay. To achieve similar concentrations of synthetic Aβ and CSF/serum-borne Aβ, standard curves of all synthetic Aβ was generated and the subject's CSF and serum Aβ oligomers values were determined by comparison to the appropriate standard curve. MonoAβ_1–40_, monoAβ_1–42_, scramAβ_25–35_, and sub-serum displayed no toxicity toward RA-SH-SY5Y neuroblastoma cells as compared to untreated cells (*p* ≤ 0.05) (Figure 4). In contrast, treatment with oligoAβ_1–40_, oligoAβ_1–42_, fibAβ_1–40_, fibAβ_1–42_, and sub-CSF lead to significant cell death as compared with untreated cells, resulting in ≤61% cell viability for treatment with both oligoAβ_1–40_ and fibAβ_1–40_ (*p* ≤ 0.05) and ≤44% cell viability for treatment with oligoAβ_1–42_, fibAβ_1–42_, and sub-CSF (*p* ≤ 0.05) (Figure 4).

Cell viability was significantly affected following treatment with oligoAβ_1–42_ compared with treatment with the fibrillary species of Aβ_1–42_ (17 vs. 27%; *p* ≤ 0.05), while treatment with sub-CSF lead to 44% cell death. These results show that the subject's CSF induced RA-SH-SY5Y cell death and confirmed the potent toxic effects of Aβ soluble oligomers previously shown to affect neurons (Bate et al., 2008).

### CSF but not serum derived from the subject accelerates Aβ aggregation kinetics *in vitro*

**Figure 5:**
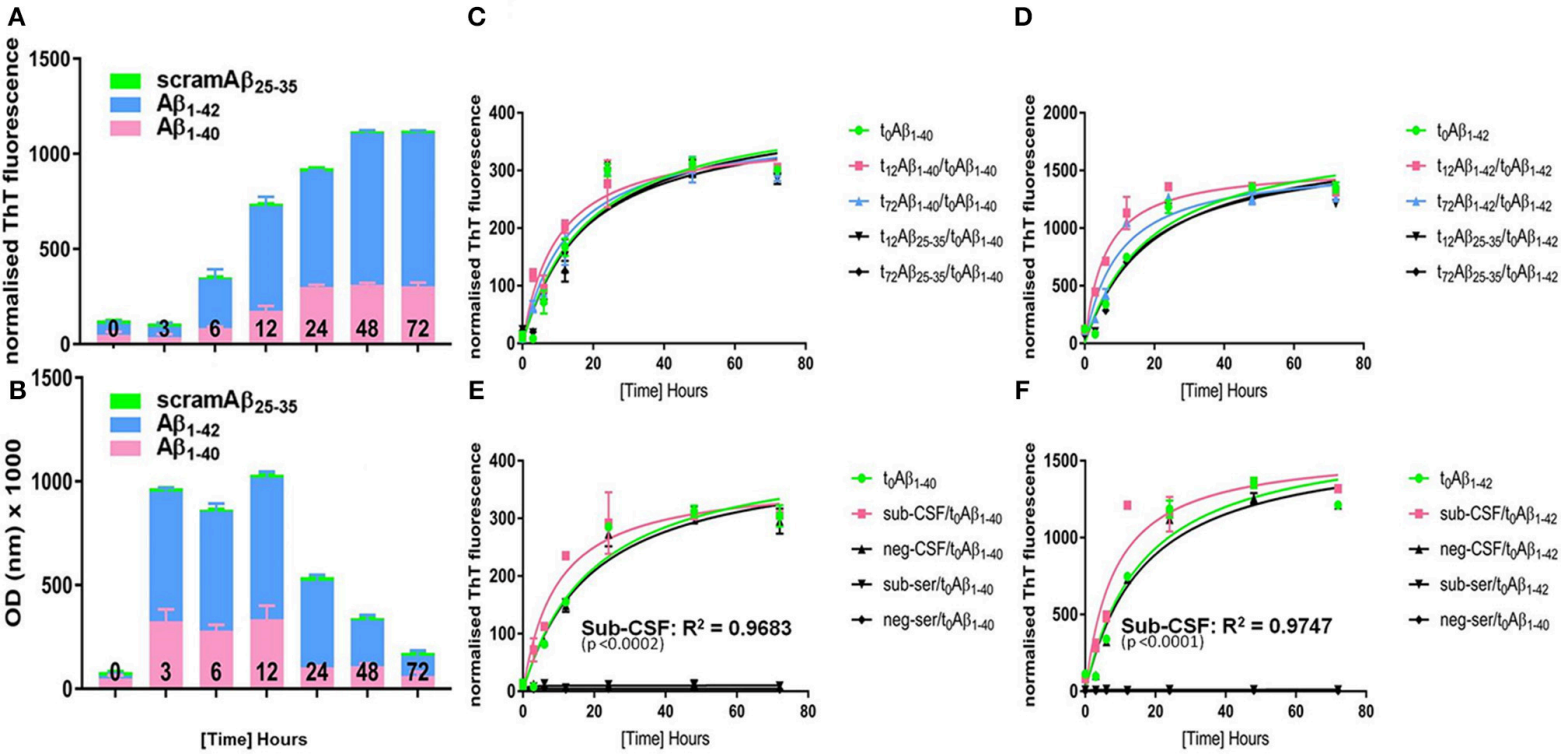
Aggregation kinetics of “seed-free” synthetic monomeric Aβ peptide. Synthetic seed-free Aβ monomers were used to produce **(A)** PRIOC10-labeled Aβ soluble oligomers and **(B)** Aβ ThT-labeled fibrils in a kinetic reaction (0–72 h). *t*_0_ is the initial time just before starting the kinetic reaction (*t*_0_ = seed-free monomers only); *t*_12_ represents maximal OD value of PRIOC10-specific Aβ oligomers (*t*_12_ = maximal oligomer yield); and *t*_72_ represents maximal value of ThT-specific Aβ fibrils (*t*_72_ = maximal fibrils yield). **(C)** seed-free Aβ_1–40_ (*t*_0_) aggregation kinetic was assessed after addition of known quantities of preformed Aβ_1–40_ amyloid seeds (*t*_12_ and *t*_72_) and compared with the aggregation kinetic after addition of conversion buffer or scrambled Aβ_25–35_ (*t*_12_ and *t*_72_). **(D)** seed-free Aβ_1–40_ (*t*_0_) aggregation kinetic was assessed after addition of known quantities of preformed Aβ_1–42_ amyloid seeds (*t*_12_ and *t*_72_) and compared with the aggregation kinetic after addition of conversion buffer or scrambled Aβ_25–35_ (*t*_12_ and *t*_72_). **(E)** seed-free Aβ_1–40_ (*t*_0_) aggregation kinetic was assessed after addition of known quantities of sub-CSF and sub-serum and compared with the aggregation kinetic after addition of conversion buffer, negative control CSF (neg-CSF) or serum (neg-serum). **(F)** seed-free Aβ_1–42_ (*t*_0_) aggregation kinetic was assessed after addition of known quantities of sub-CSF and sub-serum and compared with the aggregation kinetic after addition of conversion buffer, negative control CSF (neg-CSF) or serum (neg-serum). Error bars represent the mean level derived from n = 4 wells.

We first demonstrated that PRIOC10 immunodetected Aβ soluble oligomer species derived from monoAβ_1–40_ and monoAβ_1–42_ peptides (Figure 5A). Secondly, ThT fluorescence intensity of the fibrilar species was measured following conversion of monoAβ_1–40_, monoAβ_1–42_, and scramAβ_25–35_ peptides was assessed and was shown to be inversely proportional to levels of PRIOC10-specific oligomer species (Figure 5B). ThT did not bind to scramAβ_25–35_ peptide before and after being incubated in conversion buffer and to monoAβ_1–40_ and monoAβ_1–42_ peptides before conversion. Of note and as shown previously, PRIOC10 failed to bind the fibrilar species (Tayebi et al., 2011).

We then assessed aggregation kinetics of “seed-free” synthetic monomeric Aβ peptide following addition of Aβ oligomers or fibrils (Figures 5C,D). A known concentration of Aβ prepared by conversion during 12 h (*t*_12_) and 72 h (*t*_72_), as described above, was used in the seeding reaction; as *t*_12_ represents maximal optical density (OD) expression of Aβ soluble oligomers immunodetected with PRIOC10 (Figure 5A) and *t*_72_ represents maximal fluorescence expression of Aβ fibrils bound to ThT (Figure 5B).

Here, we added 10 pmol oligoAβ_1–40_/oligoAβ_1–42_ (*t*_12_), fibAβ_1–40_/fibAβ_1–42_ (*t*_72_), or scramAβ_25–35_ (*t*_12_ and *t*_72_) prepared by conversion during *t*_12_ and *t*_72_ to 2 mM monoAβ_1–40_, or monoAβ_1–42_ in order to assess their effects on the “*lag-phase*” kinetic as measured by ThT fluorescence. We report that *t*_12_ oligoAβ_1–40_, oligoAβ_1–42_ but not post-conversion scramAβ_25–35_ led to substantial reduction of the “*lag-phase*” (*p* ≤ 0.05) compared to *t*_0_ Aβ (Figures 5C,D). Importantly, we show that oligoAβ_1–42_ was more efficient in shortening the “*lag-phase*” compared to oligoAβ_1–40_ (*p* ≤ 0.05). Both *t*_72_ fibAβ_1–40_ and fibAβ_1–42_ but not post-conversion scramAβ_25–35_ affected the Aβ aggregation kinetic by shortening the reaction's “*lag-phase*,” albeit the effect was limited when compared to the addition of the oligomers (Figures 5C,D), reflecting a weaker seeding ability of the fibrils.

Finally, the subject's serum and CSF was added to “seed-free” synthetic monomeric Aβ to investigate whether pre-existing oligomer seeds contained in the serum and CSF of the aged dog can affect the Aβ aggregation kinetics through reduction of the “*lag-phase*” and to explore if cross-species interaction of dog Aβ with human Aβ synthetic peptide overcomes the so-called “species barrier” as applies for prion disorders (Hill and Collinge, 2002). First, we tested whether the precipitation protocol altered the conformation of Aβ soluble oligomer content of sub-CSF and sub-serum. We show that PRIOC10-specific Aβ oligomers in TCA/acetone treated sub-CSF and sub-serum were preserved (Figure S1). Total protein (20 μl of 10 mg/ml) derived from sub-serum and sub-CSF samples were incubated with solutions of monoAβ_1–40_, and monoAβ_1–42_ peptides. Surprisingly and for the first time, we show that the dog's CSF led to a substantial shortening of the reaction's “*lag-phase*” (*p* ≤ 0.05) and acceleration of human synthetic monomeric Aβ_1–40_ and Aβ_1–42_ aggregation as compared to a negative control CSF derived from the Rottweiler or scramAβ_25–35_ (Figures 5E,F). Sub-CSF was more efficient in speeding up Aβ_1–42_ then Aβ_1–40_ aggregation. In contrast, sub-serum and negative control serum led to complete inhibition of the Aβ kinetics and the “lag-phase” was not observed (Figures 5E,F). Of note, similar concentrations of synthetic Aβ and CSF/serum-borne Aβ were used.

## Discussion

The neuropathological changes observed in the 12-year-old Samoyed dog were previously described in aged dogs (Youssef et al., 2016). The extra-cellular diffuse Aβ deposits were observed throughout the cerebral cortex I-IV layers adhering to the typical staged distribution recognized in cognitively impaired dogs (Pugliese et al., 2006) and human AD (Schmidt et al., 2015). Several blood vessels of the cerebral cortex displayed severe and pronounced CAA. Colle et al. have previously shown that Aβ_1–42_-positive and Congo red-Aβ_1–40_-negative deposits were predominant in the brain parenchyma of aged dogs while Aβ_1–40_ deposited to the vasculature (Colle et al., 2000). White matter degeneration was also evident in our aged dog with vacuolation of myelinated tracts, accumulation of what appears to be lipofuscin-filled macrophages as perivascular aggregates and widespread microglial activation and gliosis. In human AD, the significance of white matter degeneration remains in dispute as its significance in disease pathogenesis remains uncertain (Ihara et al., 2010) mainly because these are considered as geriatric changes and recognized in cognitively normal individuals and dogs (Lintl and Braak, 1983; Chambers et al., 2012). Notably, in our behaviorally impaired dog, we have not been able to detect *APP, PSEN1*, or *PSEN2* gene autosomal dominant mutations which are known to directly influence accumulation of Aβ plaques and CAA in human AD (Selkoe, 2001).

In human AD, Aβ soluble oligomers are considered the neurotoxic species with the ability to affect cognitive ability and alter synaptic functions (Selkoe, 2002). Attempts to detect putative relatively low amount of CSF- and serum-borne Aβ soluble oligomers in AD patients have been hampered by the lack of assays with sufficient sensitivity and specificity (Bruggink et al., 2013; Herskovits et al., 2013; Hölttä et al., 2013; Tai et al., 2013; Savage et al., 2014). The importance of Aβ soluble oligomers in the pathogenesis of cognitive deficits and their effects on synaptic activity and function have not been investigated in aged dogs with cognitive deficits; however a report by Head et al. demonstrated that levels of CSF-borne Aβ soluble oligomers correlated inversely with total brain amounts of Aβ in aged beagles (Head et al., 2010). However, a recent report by Borghys et al. showed that high levels of Aβ in the CSF of young and middle-aged dogs were detected and correlated with impaired learning (Borghys et al., 2017). The report does not specify whether Aβ measured in the CSF of these animals contains soluble oligomer species (Borghys et al., 2017). We have shown here that our anti-oligomer antibody displayed oligomer-specific bands ranging from 90 to 200 kDa in the CSF but not in serum derived from the behaviorally impaired dog, the latter probably reflecting low levels of Aβ soluble oligomer concentrations in blood (Santos et al., 2008). Similarly, Aβ was detected with our two-site sELISA in CSF; using PRIOC10 as immunocapture antibody and a biotinylated anti-Aβ (4G8), anti-Aβ_1–40_ (PrioAD12), or anti-Aβ_1–42_ (PrioAD13) for immunodetection, and further confirms the Aβ specificity of the soluble oligomers observed on western blotting. Of importance, the sELISA detected significantly higher levels of total Aβ oligomers (PRIOC10 vs. 4G8), followed by Aβ_1–42_ soluble oligomers (PRIOC10 vs. PRIOAD13 combination) then Aβ_1–42_ soluble oligomers (PRIOC10 vs. PRIOAD12). In contrast with the result observed on Western blotting, we were able to detect soluble oligomers in the serum of our aged dog, albeit the levels were much lower than those observed in the CSF. Taken together, these results demonstrate the presence of different species of Aβ soluble oligomers in the subject's CSF in agreement with the results reported by Head et al. (2010). The results also confirm the “hierarchy” of Aβ_1–42_ over Aβ_1–40_ as observed in human AD (Armstrong, 2014).

We then set out to investigate the toxic nature of CSF- and serum-borne Aβ species derived from our behaviorally impaired dog by treating a neuron-like cell line and compare their effects with synthetic Aβ oligomers and fibrils derived from synthetic monomeric Aβ_1–40_ and Aβ_1–42_. Surprisingly, CSF but not serum derived from our behaviorally impaired dog significantly affected cell viability as measured by MTT. Several studies have reported that soluble oligomers accumulate in the CSF of AD patients and exhibit putative neurotoxic effects of homologous Aβ *in vitro* (Bate and Williams, 2007; Shankar et al., 2007; Bate et al., 2008). To our knowledge, this is the first report showing that CSF-borne Aβ oligomers from a dog with behavioral impairment affected viability of human-derived neuron-like cell lines; further proving the potent neurotoxic effects of Aβ soluble oligomers, although other factors might be implicated in the observed toxic effect. The impact on cell viability of the CSF was compared to synthetic Aβ_1–40_ and Aβ_1–42_ oligomers and fibrils following treatment of the neuroblastoma cell line; CSF was shown to be more toxic than either Aβ_1–40_ oligomers and fibrils but expectedly less so than Aβ_1–42_ oligomers and fibrils. This is similar to our previous results showing that Aβ_1–42_ was more potent at damaging neuronal synapses compared with Aβ_1–40_ peptide (Bate et al., 2008). It is important to note that the oligomeric/fibrillar Aβ content of the CSF derived from the behaviorally impaired dog is not known, hence a more accurate comparison can only be achieved through subjecting the cells to similar and known concentration of Aβ species in biological fluids.

A central feature in AD is fibril biogenesis leading to senile plaques (Powers and Powers, 2008). The molecular mechanism underlying the formation of fibrils controls the speed and the degree of its formation and the kinetics of oligomers and protofibrils (Jarrett and Lansbury, 1993; Caughey and Lansbury, 2003) and directly influences AD pathogenesis. Here, we investigated the seeding efficiency of CSF- and serum-borne Aβ oligomers and their influence on human synthetic Aβ peptides. Initially, we established assay conditions and reproducibility by adding known concentrations of post-conversion *t*_12_-Aβ and *t*_72_-Aβ oligomers and fibrils into the reaction. As anticipated, we show that all forms of post-conversion Aβ, except scrambled Aβ_25–35_ peptide, led to a substantial reduction of the lag phase (oligoAβ_1–42_ > oligoAβ_1–40_ > fibAβ_1–_42 ≥ fibAβ_1–40_). Following addition of CSF derived from our behaviorally impaired dog, the Aβ aggregation kinetic was substantially altered and led to reducing the lag phase, confirming both the presence but also the potent effect of soluble oligomers. In contrast, serum, negative control CSF and scrambled Aβ_25–35_ peptide did not affect the kinetic of the reaction. The ability of CSF-borne soluble oligomer to affect synthetic peptides derived from human β sequences was perhaps expected as the canine APP shares about 98% homology with human APP. Nevertheless, this result is important as it demonstrates inter-species molecular interaction but does not suggest nor does it demonstrate any interspecies transmission between humans and dogs. Finally, studies are underway to determine whether the above findings are a more universal phenomenon recognized in different breeds of aged and behaviorally impaired dogs.

## Concluding remarks

We have comprehensively demonstrated that this behaviorally impaired dog exhibited Aβ and Aβ soluble oligomers in its blood, CSF, and brain. We also show that the dog's Aβ affects the survival of human-derived neuron-like cell line and has a direct effect on the aggregation kinetics of human synthetic Aβ peptides. The study failed to demonstrate the involvement of phospho-tau and more genetic and molecular studies are needed to decipher its role in the neuropathology underlying cognitive dysfunction, yet we would advocate that dogs with behavioral impairments should be studied and the disease mechanisms investigated in a similar fashion as is the case with AD.

## Author contributions

CR: Performed Clinical assessment of the dog, performed MRI and revised manuscript; FS: Performed experiments; MD: Performed experiments; KF: Performed experiments; JB: Performed experiments; RG: Performed experiments; AR-L: Performed experiments; DG: Performed experiments; EH: Conceived experiments and revised manuscript; SB: Conceived experiments and revised manuscript; BS: Conceived experiments and revised manuscript; JH: Conceived experiments and revised manuscript; MT: Designed experiments, performed experiments and wrote manuscript.

### Conflict of interest statement

The authors declare that the research was conducted in the absence of any commercial or financial relationships that could be construed as a potential conflict of interest.
