# Supplementary material for: An Aged Canid with Behavioral Deficits Exhibits Blood and Cerebrospinal Fluid Amyloid Beta Oligomers
Source: Front Aging Neurosci. 2018 Jan 30;10:7. doi: 10.3389/fnagi.2018.00007 (PMC5797595; doi:10.3389/fnagi.2018.00007)
Supplement: Supplementary file 1 [file Table1.DOCX]

| **Antibody** | **Dilution** | **Source** | **Pre-treatment** | **Primary Antibody incubation** | **Secondary Antibody**  **(1:200)** | |
| --- | --- | --- | --- | --- | --- | --- |
|  | | | | | **Swine anti Rabbit**  **Dako E0353** | **Rabbit anti Mouse**  **Dako E0354** |
| **p62** | 1:500 | Abcam ab91526 | CC1-90min | 6h | 1h | - |
| **Aβ** | 1:50 | DAKO M0872 | Formic Acid-15min | 8h | - | 1h |
| **AT8** | 1:1000 | Innogenetics 90206 | CC1-90min | 1h | - | 32min |
| **SMI-31** | 1:5000 | Biolegend 801601 | none | 15min | - | 32min |
| **SMI-94** | 1:500 | Covance SMI94-R | CC1-90min | 32min | - | 32min |
| **GFAP** | 1:1000 | Dako Z0334 | Protease 1-4min | 32min | 32min | - |
| **Iba1** | 1:250 | Wako 019-19741 | CC1-45min | 1h | 32min | - |
| **SMI-94** | 1:500 | Covance SMI94-R | CC1-90min | 32min | - | 32min |
| **NF200** | 1:200 | Sigma N5389 | Protease 1-4min | 32min | - | 32min |

**Table 1** Antibodies and antigen demasking
