# Supplementary material for: An Aged Canid with Behavioral Deficits Exhibits Blood and Cerebrospinal Fluid Amyloid Beta Oligomers
Source: Front Aging Neurosci. 2018 Jan 30;10:7. doi: 10.3389/fnagi.2018.00007 (PMC5797595; doi:10.3389/fnagi.2018.00007)
Supplement: Supplementary file 2 [file Table2.DOCX]

| Gene | Exon | Forward primer | Reverse primer | Reaction | PCR cycle | Annealing temperature |
| --- | --- | --- | --- | --- | --- | --- |
| *APP* | 1 | GGCAGGAGAGAGCAGCAG | TCCAGATACAAGGCTCGGAG | 3 | GC-rich | see Material and methods |
|  | 2 | TGTTCCATTGTCAAGTTCCTCT | AGCCAAGATTTTCACAGCCA | 1 | TD | AT1 = 65°C; AT2 = 55°C |
|  | 3 | CTGGATGATAGCAGTGCCTT | AGGCCTTTTCATTCAGCAGT | 1 | TD | AT1 = 65°C; AT2 = 55°C |
|  | 4 | CCCATCTTTCCTTGATACCAACC | ACCTGGTTTTGACTCCTGAAA | 1 | CT | AT = 50°C |
|  | 5 | CCCACTCAAAACAGCAAATGG | ACATTAACAAGGGTGGGCAG | 1 | CT | AT = 59°C |
|  | 6 | TCGATACCACATTCCCTCTCA | CTGGTTGGGGAAGCCTGT | 1 | TD | AT1 = 65°C; AT2 = 55°C |
|  | 7 | TAGCGCCTAAGACAGACAGG | AGTTAGAGGAGCGCACAGAG | 1 | TD | AT1 = 65°C; AT2 = 55°C |
|  | 8 | GGTTTACTTTGGTCCCCTGC | TGGAAAGACAGGTGTTCGAGA | 1 | CT | AT = 52°C |
|  | 9 | CCGTGCTGTGAAATGTCTCA | CTAGGCAGACTCAGAGGCAG | 1 | TD | AT1 = 65°C; AT2 = 55°C |
|  | 10 | TGGAGATAGGACTGGAATGTGA | ACAATGATAAGCCATAGTCCAGA | 1 | CT | AT = 57°C |
|  | 11 | TGGAGTACAAGAAAGGGAGGA | AGACAAGATGAGCTGAGGGG | 1 | TD | AT1 = 65°C; AT2 = 55°C |
|  | 12 | ACCCATTAAGACAGAGGTGAGA | TCAGAAAATGTCAAGCCACAGAA | 1 | CT | AT = 50°C |
|  | 13 | ATCTGTCTGTGGTGCTTGGT | TGCTGCAGACACACTTAGGT | 1 | TD | AT1 = 65°C; AT2 = 55°C |
|  | 14 | TCAAATCATGTCTGGCAGGC | GCCACCGCTACCTAAACATAC | 1 | CT | AT = 61°C |
|  | 15 | TCACATGCTTTTCCCTGCTC | AACTGCAGTGTAGACAGGCT | 1 | TD | AT1 = 65°C; AT2 = 55°C |
|  | 16 | AGCCTTTGTGTTACAGCGTT | CCTTCACTGGATTTCTGGCAC | 2 | CT | AT = 50°C |
|  | 17 | GCACCGAAGAATGGAGTGAC | TTAATGCCAAGCAAAATCTCTC | 2 | CT | AT = 60°C |
|  | 18 | CCTGCGCTCCTTATCCCTTA | GAAGGGTTTGTTTCTTTCCACA | 1 | TD | AT1 = 65°C; AT2 = 55°C |
| *PSEN1* | 1 | TAATCTGGGAGCCTGCAAGT | CTCAGTCCCCTCAGTCTCCA | 1 | TD | AT1 = 65°C; AT2 = 55°C |
|  | 2 | AGAACTCCAGTAATGATGGGTCT | AACTCTCTAGCCTTCGTGCC | 1 | TD | AT1 = 65°C; AT2 = 55°C |
|  | 3 | TCTTGGCTAGATTGCTGAGA | AGGCCCTTTTCTCTACTAGA | 2 | CT | AT = 52°C |
|  | 4 | CCAAAGAGTTCCAATATAAGTGTG | GTATTGGGATCCTGGGTGG | 1 | TD | AT1 = 60°C; AT2 = 50°C |
|  | 5 | ACAGCACAGTTTGATATAAGA | ACTGAAGAGCTATGGGATGT | 2 | CT | AT = 52°C |
|  | 6 | TCCCTTGGTTAATTCTTCCCT | CAAACAGGGATGTGGAGAAG | 2 | CT | AT = 58°C |
|  | 7 | TTTTGTAGAAAGATAATGACCTG | GTCCATGACCAAAGACAGAG | 1 | CT | AT = 52°C |
|  | 8 | TGTGACAGTTGGGTAGTTAC | CCCACTAAAAGGTACACTCTG | 1 | CT | AT = 52°C |
|  | 9 | CAGTATTAGGATGCAATACAGC | CAGCTGGGGTTAAAATGGAC | 2 | CT | AT = 55°C |
|  | 10 | GGACTTGTGATTGGGTTGTT | TGTCCCTCAAATCTGGTAGT | 2 | CT | AT = 55°C |
| *PSEN2* | 2 | CTGTGTCCAGGTCTTGTTGC | TGACCGGTTCTCTCCCTTAG | 1 | TD | AT1 = 65°C; AT2 = 55°C |
|  | 3 | GCCCATGTCCACTTCCAC | GTAGCAACCAGCCCAGCTCT | 1 | TD | AT1 = 65°C; AT2 = 55°C |
|  | 4 | AGGACTAGGGTGGGAGCCT | GAACTGAGGCGTCTGCTCTG | 1 | TD | AT1 = 65°C; AT2 = 55°C |
|  | 5 | TGCTTGGACCTGAAGGATTT | GGCCCAAAGACTGGAATCTC | 1 | TD | AT1 = 65°C; AT2 = 55°C |
|  | 6 | GAAACAGGGGATGTTGCACT | CCCCTTGTATCCACCAGTGA | 1 | TD | AT1 = 65°C; AT2 = 55°C |
|  | 7 | TGGGACACACTCTGAGCCT | CCACCTCCTCCCCTTTAATC | 1 | TD | AT1 = 65°C; AT2 = 55°C |
|  | 8-9 | TGTCTTCATGTGGAGGCAAA | CTGATGTGAAGAGGGGCTTC | 1 | TD | AT1 = 65°C; AT2 = 55°C |
|  | 10 | GGCTGTGGACTGGTGTCTTC | GGCAGCAGCATCTGAAACTT | 1 | TD | AT1 = 65°C; AT2 = 55°C |
|  | 11 | CTGTTGTCTGACCCACCTGA | CGGTTCCTACGGATAGACCA | 1 | TD | AT1 = 65°C; AT2 = 55°C |

**Table 2** PCR conditions used for each primer set
