# Supplementary material for: An Aged Canid with Behavioral Deficits Exhibits Blood and Cerebrospinal Fluid Amyloid Beta Oligomers
Source: Front Aging Neurosci. 2018 Jan 30;10:7. doi: 10.3389/fnagi.2018.00007 (PMC5797595; doi:10.3389/fnagi.2018.00007)
Supplement: Supplementary file 3 [file Presentation1.PDF]

Materials and Methods:

Clinical Investigations:

A 12-year-old neutered male Samoyed was presented to a specialist veterinary practice (Fitzpatrick Referrals, UK) for pain management and evaluation of difficulty rising. Initial evaluation by the referring veterinarian had included a whole-body CT with routine haematology and biochemistry. After ascertaining that the behavioural changes reported by the owner suggested cognitive function deficits as opposed to pain, the patient was investigated with brain MRI using a 1.5T system (Siemens Symphony, Erlangen, Germany) The MRI scan for was performed with dog positioned in dorsal head first on the scanning couch. The MRI protocol of the head consisted with following sequences and parameters: T2 Weighted (W) in sagittal (TR 3220ms, TE 109ms, Slice thickness 3.2mm, Averages 3, matrix 384x320, FOV 180mm), T2W in transverse (TR 3600ms, TE 112ms, Slice thickness 4mm, Averages 2, matrix 320x320, FOV 150mm), T1W in transverse (TR 400ms, TE 11ms, Slice thickness 4mm, Averages 3, matrix 384x320, FOV 150mm), Fluid Attenuation Inversion Recovery (FLAIR) in transverse plane (TR 8000ms, TE 123ms, Slice thickness 4mm, Averages 2, matrix 512x512, FOV 150mm), T2 Gradient Echo in transverse (TR 537ms, TE 17ms, Slice thickness 4mm, Averages 2, matrix 512x512, FOV 150mm), post contrast T1W in transverse and dorsal planes (TR 400ms, TE 11ms, Slice thickness 4mm, Averages 3, matrix 384x320, FOV 150mm). In the imaging examination it was included overview of the entire spinal column in sagittal T2W (TR 3400, TE 100ms, slice thickness 2.5mm, averages 4, matrix 512x512, FOV 330mm) and Short-tau Inversion Recovery in dorsal plane (TR 3400ms, TE 54ms, slice thickness 3.5mm, averages 2, matrix 384x320, FOV 360mm, as well as the sagittal plane of both shoulders using Proton Density (PD) sequence (TR 2830ms, TE 12ms, Slice thickness 3mm, averages 2, matrix 320x320, FOV 220mm. Following imaging, a cisternal cerebrospinal fluid (CSF) and elbow and shoulder joint fluid samples were obtained for cytological assessment and protein measurement. Blood pressure was assessed non-invasively using a Doppler ultrasound probe when the dog was conscious and fully recovered from anaesthesia. Twenty measurements were made over 2 consecutive days to improve confidence that results did not reflect anxiety. Retinal examination was performed using an ophthalmoscope. The dog was also investigated with routine haematology, serum and urine biochemistry, and urine culture.

Histopathology and Immunohistochemistry:

At the owner's request, the subject was euthanized at 12 years of age and a full post-mortem examination was conducted. The subject's brain was sectioned into two halves; the left hemisphere was frozen at -80C and the right hemisphere was fixed by immersion in 10% neutral buffered formalin. Tissue slices from different anatomical regions of the cerebrum and cerebellum were then processed through graded alcohols and xylene and embedded in paraffin. The tissues were sectioned at 4μm for staining with haematoxylin and eosin (H&E) or immunohistochemistry (IHC). IHC was performed on an automated Ventana Discovery XT instrument (Roche, Burgess Hill UK), using the Ventana DAB Map Detection Kit (760-124). Details of antibodies used and epitope demasking protocols are described in Table 1. For pre-treatment either Ventana Protease 1 (760-2018), Ventana CC1 (950-124), equivalent to EDTA buffer, or Formic Acid (VWR, 20320.320) was used. Slides were counterstained with haematoxylin, dehydrated and cover-slipped using Pertex (Celltech) as a mounting. Sections were then viewed using a light microscope (Nikon Eclipse) and scanned using a LEICA SCN400 slide scanner. Digital images were taken from e-slides using LEICA Slidepath image management and viewing software.

Genetic analysis of the subject's presenilin 1, presenilin 2 and amyloid precursor protein genes:

DNA was extracted from blood using a QIAamp DNA Blood Kit (Qiagen, Manchester, UK) as per manufacturer's instructions. All coding exons of presenilin 1 (*PSEN1*) 1, presenilin 2 (*PSEN2*) and amyloid precursor protein (*APP*) genes were amplified and Sanger sequenced. Three types of PCR reactions were used depending on the primers. Reaction 1 consisted of 0.8x FastStart PCR Master mix (Roche, Welwyn Garden City, UK), 4% DMSO and primers were at 0.4 μM each in a total of 25 μl. Reaction 2 consisted of 1x Buffer, 1x Q-Solution, 200 μM of each dNTP, 0.4 μM of each primer and 1.25 U of *Taq* DNA Polymerase (Qiagen, Manchester, UK) in a total of 30 μl. Reaction 3 consisted of 1x FastStart PCR Master mix (Roche, Welwyn Garden City, UK), 7% DMSO, 1.8x Q-Solution, 0.18 mM deazaGTP, 0.9 mM $MgCl_2$ and 0.36 μM of each primer in a total of 14 μl. Samples prepared in Reaction 1 and 2 were amplified in one of two different PCR cycles. The touchdown (TD) PCR cycle comprised an initial denaturation step at 95°C for 5 min; 20 cycles of 95°C for 15 s, 30 s on the annealing temperature 1 (AT1) that decreased 0.5°C for every cycle, and 72°C for 30 s (except *PSEN2* exon 8-9: 45 s); extra 10 cycles of 95°C for 15 s, annealing temperature 2 (AT2) for 30 s, and 72°C for 30 s (except *PSEN2* exon 8-9: 45 s); and a final extension step at 72°C for 5 min. The constant (CT) PCR cycle consisted of an initial denaturation step at 94°C for 5 min; 30 cycles of 94°C for 30 s, constant annealing temperature (AT) for 30 s, and 72°C for 30 s; and a final extension step

at 72°C for 5 min. The sample prepared in Reaction 3 was amplified with a GC-rich PCR cycle of an initial denaturation step at 96°C for 3 min; 20 cycles of 95°C for 1 min, 72°C for 30 s with a touchdown decrease of 0.5°C for every cycle, and 69°C for 45 s; 10 cycles of 95°C for 1 min, 60°C for 30 s, and 69°C for 45 s with a time increment of 5 s in every cycle; extra 20 cycles of 95°C for 1 min, 60°C for 30 s with a touchdown decrease of 0.5°C for every cycle, and 69°C for 45 s with a time increment of 5 s in every cycle; and a final extension step at 69°C for 7 min. Table 2 summarizes the PCR conditions used for each primer set. Amplification was confirmed by electrophoresis of the PCR products using a 1.5% agarose gel. After confirmation, PCR products were cleaned-up with a mixture of 0.2 U Fast-AP (Thermo Scientific, Leicestershire, UK) and 1 U Exonuclease I (Thermo Scientific, Leicestershire, UK) in water. Sequencing PCR consisted of 0.9x Sequencing Buffer, 0.12x of BigDye Terminator v3.1 Ready Reaction mix (ABI, Bleiswijk, Netherlands), 0.9 µM of one of the primers (all exons were sequenced using the forward and reverse primers) and 5 µl of PCR product in a total of 11 µl. Purification of the sequencing PCR was performed with the Dye Terminator Removal Kit (Thermo Scientific, Leicestershire, UK) as per manufacturer's instructions. Samples were run in a 3730xl DNA Analyzer and Sanger sequencing results were analysed with Sequencher™ (Gene Codes Comporation, Ann Arbor, USA).

SDS polyacrylamide gel electrophoresis (SDS-PAGE):

SDS-PAGE pre-cast gels (Invitrogen) were used. The subject's serum (sub-serum) and CSF (sub-CSF) samples to be electrophoresed were diluted 1:1 in 40 µl sample buffer and boiled for 5 minutes in screw-cap Eppendorf tubes. The samples were spun for 5 seconds at 14.000 rpm in a microfuge before being loaded on precast 6–18% polyacrylamide gels. The gels were electrophoresed at a constant voltage of 200V for 1 hour. Following electrophoresis, gels were blotted onto Invitrolon PVDF (Invitrogen) in the POWER-PAC (BIO-RAD), at 18V for 45 minutes. Following blotting, the membranes were rinsed in PBS-tween (0.05%) before being transferred to Odyssey Blocking Buffer (LI-COR Biosciences) for 60 minutes at room temperature. The membranes were again rinsed in PBS-tween (0.05%) to remove all traces of blocking solution. A solution of 5 µg/ml purified PRIOC antibody (14) was added and incubated for 1 h at room temperature. Following 4 washes of 15 min each, the membranes were then incubated in IRDye 800 CW goat, anti-mouse IgM secondary antibody (1:10,000; LI-COR Biosciences) for one hour at room temperature. The membranes were washed as above, and imaged using an

Odyssey CLx infrared imaging system (LI-COR Biosciences), according to the manufacturer's instructions. Signal development times ranged from 1 second to 30 minutes. All experiments were repeated at least three times.

Sandwich ELISA for detection of soluble oligomers:

Medium binding, 96 well plates (Greiner) were coated with 50 µl/well of a 5 µg/ml purified PRIOC10, 1 µg/ml 4G8 (Aβ), PRIOAD12 ($Aβ_{1-40}$) or PRIOAD13 ($Aβ_{1-42}$) (16, 17) antibody solution in coating buffer. The plates were incubated for 1 hour at 37°C then washed 3 times with PBS-0.05% tween, and then blocked for 1 hour at room temperature. Sub-serum and sub-CSF diluted 1:1 in 0.5% in PBS-0.05% tween 20 (w/v) with protease inhibitors (Roche Biochemicals) was added and incubated for 1 hour at 37°C. The plates were then washed 3 times with PBS-0.05% Tween (Sigma) and a 5 µg/ml of biotinylated PRIOC10 antibody was added for 1 hour at 37°C and the plates were again washed 3 times with PBS-0.05% tween before addition of a 1/1000 dilution of horseradish-peroxidase (HRP) conjugated streptavidin (Sigma) for 25 minutes at 37°C and the plates were again washed 4 times with PBS-0.05% tween. Finally the plates were developed with o-Phenylenediamine dihydrochloride (OPD) buffer until optimum development occurred when the reaction was stopped with 3 M sulphuric acid prior to spectrophotometric reading (BMG LABTECH) at 490 nm. All experiments were repeated at least three times.

Assessing neurotoxic effect of serum and CSF in human neuroblastoma cell line:

Commercially available immortalised human neuroblastoma cell line, Retinoic acid (RA)-differentiated [(RA-SH-SY5Y, ECCC; Encinas et al., 2000)] were used in the studies documented here. Therefore, for these studies, no ethical approval was required for their use.

RA-SH-SY5Y were plated at 4 x 10^6 in collagen-coated culture dishes in Dulbecco's modified Eagle's medium (DMEM) supplemented with 2 mM L-glutamine, penicillin (20 units/ml), streptomycin (20 mg/ml), and 10% (v/v) heat-inactivated foetal calf serum (GIBCO). Cultures were maintained at 37°C in 5% CO_2 with a change of medium every 72 hours. For survival assays, RA-SH-SY5Y cells were plated into 48-well plate at 5 x 10^4 cells per well and left to adhere overnight prior to treatment. Wells were then incubated with either 50 µM mono$Aβ_{1-40}$, mono$Aβ_{1-42}$, scram$Aβ_{25-35}$, oligo$Aβ_{1-40}$, oligo$Aβ_{1-42}$, fib$Aβ_{1-40}$, fib$Aβ_{1-42}$ peptides as well as subject's total protein derived from sub-serum and sub-CSF samples (100 µl of 2 mg/ml) for 48 hours at 37 °C and 5% CO_2. The cells were then washed in PBS and cell survival was determined following addition of thiazolyl blue tetrazolium (25

mM, MTT). Optical density (OD) values were generated with spectrophotometric reading (BMG LABTECH) at 450 nm. All experiments were repeated at least three times.

Production of soluble oligomers and fibrils from synthetic monomeric human Aβ peptides:

The human-derived synthetic monomeric $A\beta_{1-40}$ and $A\beta_{1-42}$ peptides (purity $\geq$95% by HPLC analysis, Signal Chem) were used for the production of soluble oligomers and fibrils. The scrambled synthetic $A\beta_{25-35}$ peptide (scram$A\beta_{25-35}$; purity $\geq$95% by HPLC analysis, Signal Chem) was also used as control in the seeding experiments. Initially, $A\beta_{1-40}$, $A\beta_{1-42}$ and scram-$A\beta_{25-35}$ peptides were cleared of any potential seed aggregates that might have formed spontaneously. An aqueous solution of sodium hydroxide (NaOH, 100 mM, *pH* 10.5) was added to lyophilised $A\beta_{1-40}$, $A\beta_{1-42}$ and scram-$A\beta_{25-35}$ prior to sonication (Fisher Scientific) for 20 minutes at 25°C. The sonicated preparation was then filtered using 30 kDa filters (Millipore) in sequence**.** Following filtration, the concentration of the end-products (called mono$A\beta_{1-40}$, mono$A\beta_{1-42}$ and scram$A\beta_{25-35}$ was measured at 280 nm through absorption spectrophotometry (BMG LABTECH). Soluble oligomers and fibrils derived from their equivalent monomeric isoforms were produced as previously described (13, 14, 18). Briefly, soluble oligomers were produced by adding 1 mg of mono$A\beta_{1-40}$ and mono$A\beta_{1-42}$ peptides (as well as scram$A\beta_{25-35}$) prepared as described above into 400 µl hexafluoro-2-propanol (HFIP). The solutions (100 µl) were added to 900 µl ddH_2O and incubated for 10–20 minutes at RT, then centrifuged for 15 min at 14,000× g. The samples were then stirred at 500 rpm for 72 h at 22°C and 10 µl aliquots were frozen at 80°C until further use. The end products were called oligo$A\beta_{1-40}$ and oligo$A\beta_{1-42}$. Synthetic tau and α-synuclein proteins were also used to generate their oligomer counterparts. A customised Sandwich ELISA assay using oligomer-specific PRIOC10 antibody confirmed the presence of soluble oligomers (14). The fibrils derived from the monomeric isoforms were prepared as described previously (13) and the end products was called fib$A\beta_{1-40}$ and fib$A\beta_{1-42}$. The Thioflavin T (ThT) fluorescence intensity of the samples was recorded following incubation of 200 µl sample with ThT and confirmed presence of fibrils.

Kinetic of Aβ aggregation induced by serum and cerebrospinal fluid derived from the subject:

Aliquots (2 mM) of mono$A\beta_{1-40}$, mono$A\beta_{1-42}$, scram$A\beta_{25-35}$, oligo$A\beta_{1-40}$, oligo$A\beta_{1-42}$, fib$A\beta_{1-40}$, fib$A\beta_{1-42}$ peptides were resuspended to 200µl volumes in 0.1M Tris-HCl, *pH* 7.4. Solutions of mono$A\beta_{1-40}$ and mono$A\beta_{1-42}$ peptides were added to opaque, clear bottom 96-well microplates (Costar) and incubated in the presence of 10 pmol (v/v)

oligoAβ_{1-40}, oligoAβ_{1-42}, fibAβ_{1-40}, fibAβ_{1-42} or scramAβ_{25-35}peptides with addition of 5 mM ThT. Sub-serum and sub-CSF were mixed with the monomeric peptides to assess their effect on the aggregation kinetic. Following centrifugation of 2 ml of sub-serum and sub-CSF aliquots (800 × *g*, 5 min), supernatants were kept at −80°C until further analyses. Trichloroacetic acid (TCA)/acetone precipitation protocol was used to precipitate proteins from supernatants and total protein concentrations were measured at 280 nm through absorption spectrophotometry (BMG LABTECH). 20 μl of 10 mg/ml total protein derived from sub-serum and sub-CSF samples were incubated with solutions of monoAβ_{1-40} and monoAβ_{1-42} peptides. The microplates were sealed then loaded into a FLUOstar OMEGA (BMG LABTECH) and incubated at 37 °C for 72 hours. The ThT fluorescence was measured from the bottom of the microplates at various time points. All experiments were repeated at least three times.

Statistical Analysis: One-way ANOVA with Dunnett's post-test was performed using GraphPad Prism version 7.00 for Windows, GraphPad Software, San Diego California USA, for statistical analysis.
