# Supplementary material for: An Aged Canid with Behavioral Deficits Exhibits Blood and Cerebrospinal Fluid Amyloid Beta Oligomers
Source: Front Aging Neurosci. 2018 Jan 30;10:7. doi: 10.3389/fnagi.2018.00007 (PMC5797595; doi:10.3389/fnagi.2018.00007)
Supplement: Supplementary file 4 [file Presentation2.PDF]

**Clinical presentation:**

**History:**

A 12-year-old neutered male Samoyed dog was presented to Fitzpatrick Referrals, UK for pain management and evaluation of difficulty rising. Initial evaluation by the referring veterinarian had included a CT which had revealed multiple Hansen type II disc protrusions in the thoracic and at the lumbosacral disc spaces but without significant encroachment of nervous tissue. CT had revealed mild elbow and shoulder osteoarthritis. Pre-referral haematology suggested a mild non-regenerative anaemia; red blood cells 4.7 x10^9/ml (reference interval (RI): 5.5-8.5) with a haemoglobin of 11.5g/dl (RI: 14-20g/dl)). Serum biochemistry was unremarkable with the exception of a mild elevation of lactate dehydrogenase (543 IU/l; RI: 50-320). Serum thyroxine was normal at 22.9 nmol/l (RI: 13.0–51.0 nmol/L). The dog made a poor recovery from the general anaesthesia which had been required for the advanced diagnostic imaging and later that night was presented to the primary veterinary surgeon as emergency with behavioural changes described by the owner as extreme agitation accompanied by repetitive aimless vocalisation and pacing. This behaviour was initially interpreted as a response to pain. Consequently, and over the following week he received polypharmacy including corticosteroids, methadone, fentanyl, diazepam, gabapentin and tramadol. Following the polypharmacy, the dog became increasingly aggressive on handling and unable to rise and subsequently was referred for a second opinion.

**Clinical and neurological examination:**

During the first examination, the dog was drowsy, but once roused would pace around the examination room in a disorientated and poorly interactive manner, often staring at and getting stuck in room corners. With the exception of mild discomfort on elbow and shoulder manipulation, consistent with the previous diagnosis of osteoarthritis, no painful focus was identified. Clinical examination also suggested a urinary tract infection with haematuria and foul smelling urine. Neurological examination revealed tetraparesis and reduced spinal reflexes and muscle tone consistent with a polyneuropathy. The difficulty rising was attributed to this, complicated by the sedative polypharmacy. The historical and consulting room behaviour suggested a cognitive function deficit possibly complicated by a urinary tract infection. Retinal examination was unremarkable with no evidence of retinal blood vessel changes or papilledema.

**Further diagnostic tests:**

The brain MRI scan revealed a diffuse cortical atrophy and a hyperintensity in the white matter on T2W, particularly in the corona radiata (Fig. S1); changes consistent with age-associated cognitive decline (1).

Spinal MRI was unremarkable and confirmed the mild non-clinically significant Hansen type II disc protrusions identified in the CT study. CSF analysis was unremarkable with all parameters within the normal ranges. CSF that was excess to that required for the diagnostic tests was used subsequently in this study. Joint fluid analysis revealed changes consistent with mild osteoarthritis. Haematology confirmed a mild non-regenerative anaemia with a neutrophilia suggestive of infection (29.39 x $10^9$/L): serum biochemistry revealed mild elevation in alkaline phosphatase (262 U/L; RI: 23-212) secondary to previous corticosteroid administration. Serum that was excess to that required for the diagnostic tests was used in this study. Urinalysis suggested an infective process and culture found a heavy growth of *Proteus*. The subject was hypertensive with blood pressure ranging between 170 and 190 mmHg (normal less than 150mm/Hg). The working diagnosis was of cognitive dysfunction syndrome complicated by hypertension and urinary tract infection. It was though that the general anaesthetic and stress associated with diagnostic procedures together with the urinary tract infection had precipitated a crisis. It subsequently transpired that night-time anxiety and restlessness had been a problem for some weeks prior to the acute deterioration.

The subject was managed for the various co-morbidities as follows: cognitive dysfunction - propentofylline, selegiline and night-time alprazolam to reduce anxiety together with night time melatonin to encourage sleep; urinary tract infection – 5 day course of amoxycillin and clavulanic acid ; hypertension – amlodipine; degenerative joint disease – non-steroid anti-inflammatory drugs; polyneuropathy – L-Carnitine and omega-fatty acid supplementation. The dog made a progressive improvement over the next 8 weeks with an improvement in mobility although he still displayed regular night-time agitation and restlessness, which was controlled by alprazolam. The blood pressure normalised at 120 mmHg. Signs were controlled for the next 6 months after which the dog deteriorated described as extreme agitation and distress during the night which was unresponsive to changing dose of medication and resulting in significant sleep deprivation for the owners. After some weeks, the owners elected for euthanasia. A full post mortem examination was undertaken at the pathology facility at the University of Surrey and the brain and other organs were removed for further analysis.

1. D. Hasegawa, N. Yayoshi, Y. Fujita, M. Fujita and H. Orima: Measurement of interthalamic adhesion thickness as a criteria for brain atrophy in dogs with and without cognitive dysfunction (dementia). *Vet Radiol Ultrasound*, 46(6), 452-7 (2005)
