# Supplementary material for: An Aged Canid with Behavioral Deficits Exhibits Blood and Cerebrospinal Fluid Amyloid Beta Oligomers
Source: Front Aging Neurosci. 2018 Jan 30;10:7. doi: 10.3389/fnagi.2018.00007 (PMC5797595; doi:10.3389/fnagi.2018.00007)
Supplement: Supplementary file 5 [file Presentation3.PDF]

Immunodetection of Aβ soluble oligomers following precipitation of the subject's CSF and serum

Biotinylated PRIOC10 anti-oligomer antibody was used to immunocapture Aβ species contained in the CSF (sub-CSF) and the serum (sub-serum) precipitates with or with no treatment with trichloroacetic acid (TCA)/acetone derived from the dog. Purified PRIOC10 anti-oligomer antibody was added to immunodetect specifically Aβ soluble oligomers. The sandwich format of the assay has demonstrated the presence and preservation of the conformation of soluble oligomers following precipitation of CSF and serum with trichloroacetic acid (TCA)/acetone. Values shown are the mean Aβ ± SD from 12 observations.
