# Supplementary figures and images for: An Aged Canid with Behavioral Deficits Exhibits Blood and Cerebrospinal Fluid Amyloid Beta Oligomers
Source: Front Aging Neurosci. 2018 Jan 30;10:7. doi: 10.3389/fnagi.2018.00007 (PMC5797595; doi:10.3389/fnagi.2018.00007)

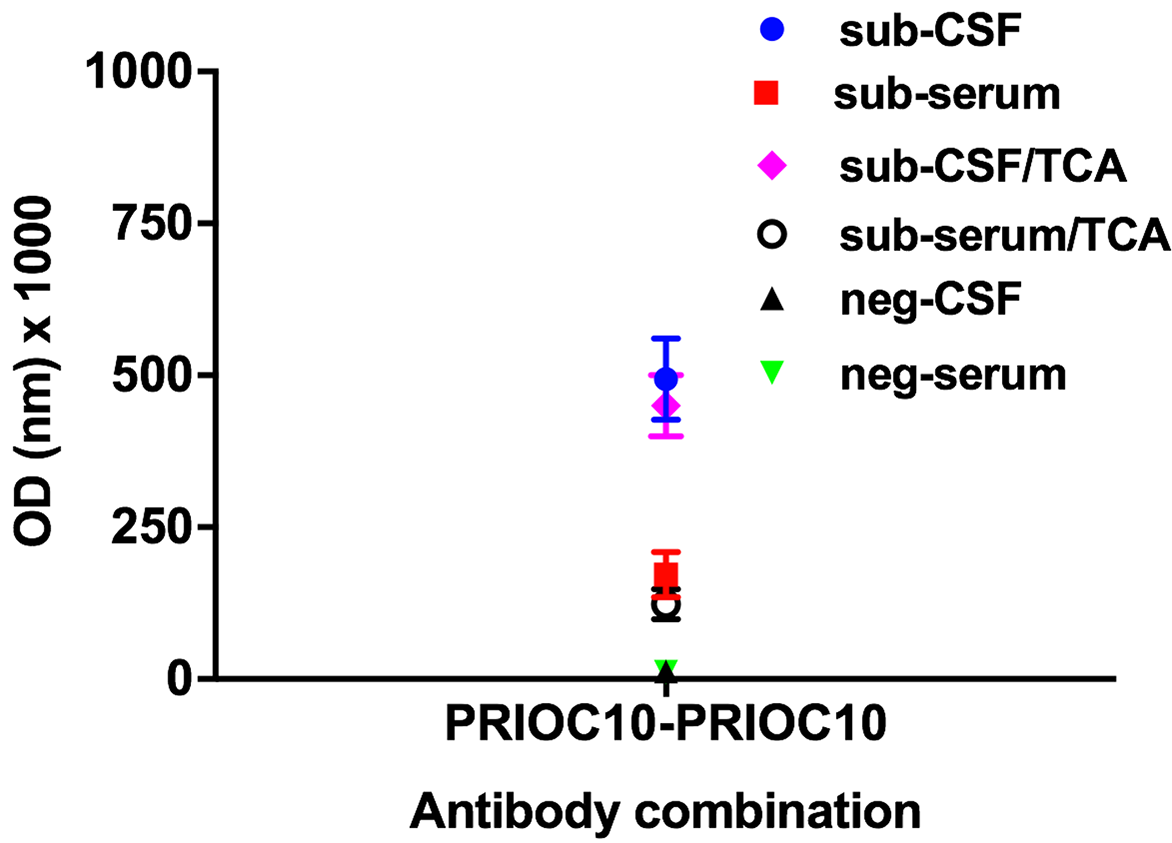

Supplement: Supplementary file 6 [file Image1.TIFF]
